# Who uses mHealth apps? Identifying user archetypes of mHealth apps

**DOI:** 10.1177/20552076231152175

**Published:** 2023-01-22

**Authors:** Maryam Aziz, Aiman Erbad, Samir B Belhaouari, Mohamed B Almourad, Majid Altuwairiqi, Raian Ali

**Affiliations:** 1 College of Science and Engineering, 370593 Hamad Bin Khalifa University, Qatar; 2 College of Technological Innovation, 54483 Zayed University, United Arab Emirates; 3 College of Computer and Information Technology, 125895 Taif University, Kingdom of Saudi Arabia

**Keywords:** Digital health, eHealth, mental health, physical health, personality, satisfaction with life

## Abstract

**Objective:**

This study aims to explore the user archetypes of health apps based on average usage and psychometrics.

**Methods:**

The study utilized a dataset collected through a dedicated smartphone application and contained usage data, i.e. the timestamps of each app session from October 2020 to April 2021. The dataset had 129 participants for mental health apps usage and 224 participants for physical health apps usage. Average daily launches, extraversion, neuroticism, and satisfaction with life were the determinants of the mental health apps clusters, whereas average daily launches, conscientiousness, neuroticism, and satisfaction with life were for physical health apps.

**Results:**

Two clusters of mental health apps users were identified using k-prototypes clustering: *help-seeking* and *maintenance* users and three clusters of physical health apps users were identified: *happy conscious occasional*, *happy neurotic occasional*, and *unhappy neurotic frequent* users.

**Conclusion:**

The findings from this study helped to understand the users of health apps based on the frequency of usage, personality, and satisfaction with life. Further, with these findings, apps can be tailored to optimize user experience and satisfaction which may help to increase user retention. Policymakers may also benefit from these findings since understanding the populations’ needs may help to better invest in effective health technology.

## Introduction

Physical and mental health saw a general decline amongst people with social isolation leading to a more sedentary lifestyle.^1^ Behavioral change support to promote better health practices is feasible using physical and mental health-based apps due to these apps being widely available and accessible.^2,3^ According to the IQVIA Institute for Human Data Science report on digital health trends, more than 350,000 consumer health apps were available to download, with most apps focused on general health and fitness.^4^ Furthermore, about 47% of apps were focused on specific health conditions, of which almost half of the apps were based on mental health, diabetes, and cardiovascular-related conditions. A survey in the US found nearly 58.3% of users had downloaded a health-related app on their mobile phones, with fitness and nutrition-based apps as the most common health app category.^5^

Digital health technologies may be used to immediately evaluate individuals’ mental and physical health, which may help reduce the burden for hospitals. A study looking into user engagement with digital health interventions found users’ interest in digital health intervention to predict their user engagement.^6^ Moreover, research also found online health-seeking behavior is related to the use of real-world healthcare; that is, people seeking online help are likely to visit hospitals and clinics for help.^7^

Personality traits have also been studied to understand the users’ adoption and engagement with digital health technologies. A review investigated the use of digital mental health interventions amongst users based on the Big-5 personality traits.^8^ The traits of neuroticism and agreeableness were often linked to users’ interest in using digital health apps for stress-related support. The extraversion trait was more linked to in-person mental health support than digital mental health interventions. The trait of openness predicted users’ higher preference for mindfulness and meditation-based interventions, and the trait of conscientiousness predicted their increased adherence to digital mental health interventions. Another study found high neuroticism and lower physical health to predict the use of digital health apps for improving personal health.^9^ The results from this study are not generalizable since they are targeted at the leaders at workplaces. However, it shows a need to understand the use of digital health apps with respect to the different psychometrics.

Personality traits often studied in research on mental health are the Big Five dimensions; extraversion, agreeableness, neuroticism, conscientiousness, and openness to experiences.^10^ Particularly, extraversion and agreeableness personality traits tend to be strongly related to positive mental health. In contrast, the personality trait of neuroticism is a strong predictor of negative mental health in individuals.^11^ According to a meta-analysis review done by Bucher et al.,^12^ personality traits were related to treatment outcomes for mental health issues, with neuroticism negatively associated with mental health treatment. The other four personality traits were found to be positively associated with mental health treatment. A study done by Osimo et al.^13^ used a survey to investigate the impact of personality traits on mental health during the pandemic. High levels of extraversion and neuroticism and low levels of openness predicted lower levels of depression and anxiety. No personality dimension was found to have affected stress levels during the coronavirus disease 2019 (COVID-19). The study depended on participants’ memory of their mental health during the first two phases of COVID-19. However, participants may not recall their mental health accurately during those times. Another study explored the personality traits of neuroticism and extraversion as predictors of mental health issues due to COVID-19 in Canada.^14^ The traits of neuroticism and extraversion are often considered important when studying mental health since people with high neuroticism and introversion are more susceptible to symptoms of depression, anxiety, and emotional distress.^15^ A study done by Klinger-König et al.^16^ found extraversion as positively related to mental health symptoms but not to physical health whereas neuroticism was negatively related to both physical and mental health symptoms.

Concerning the relationship between mental health and satisfaction with life, a survey conducted by Lombardo^17^ in Canada found a strong association between satisfaction with life and self-reported mental health using the Satisfaction With Life Scale (SWLS)^18^ for measuring life satisfaction. Furthermore, those with poor mental health reported low satisfaction levels with life.^19^ investigated the relationship of stress-related coronavirus with individuals’ satisfaction with life. Those individuals who had higher levels of COVID-related stress scored lower on SWLS. Another study investigated the relationship between depression and satisfaction with life based on gender.^20^ The results showed that depression had a significant effect on satisfaction with life, with depressed males scoring lower on SWLS than females. Their study, however, was limited since the participants were generally female, and the sample size was small, preventing the study from being generalized. Furthermore, a study investigating the continued use of a mental health wellness app aimed at stress management found that users reported improved satisfaction with life after 30 days.^21^ Their study utilized SWLS to measure the life satisfaction of the participants. However, their study used a healthy group of participants, and the results may not be the same for stressed participants.

Physical health encompasses different health aspects such as physical activity, dietary habits, and weight management.^22^ Personality traits of conscientiousness and neuroticism are often linked with physical health in literature. According to the review done by Murray and Booth^23^ on physical health and personality, higher conscientiousness predicted improved physical health while higher neuroticism tends to worsen physical health. In contrast, the personality traits of extraversion and openness sometimes positively and sometimes negatively impact physical health. A survey investigating the relationship between behavioral intentions and the use of physical health apps found gender to moderate the relationship between two of the personality traits and users’ behavioral internet to use physical health apps.^24^ The two personality traits of extraversion and neuroticism had no impact on the usage on their own but were found to be related to behavioral intention when moderated by gender. Additionally, another survey found only conscientiousness positively related to the usage of physical health apps out of the five personality traits.^25^ Stieger et al.^26^ conducted a study to understand the relationship between physical activity and the five personality traits. Their results showed that only conscientiousness was positively associated with physical activity. Additionally, a combination of higher conscientiousness and higher neuroticism was related to healthy physical activity in individuals. Being conscientious means individuals are planful and therefore, more likely to meet their physical health goals. Further, neuroticism also impacts physical health when combined with conscientiousness since theoretically individuals who are highly neurotic and conscientious would be more likely to be concerned for their physical health and plan steps to improve them^27,28^ conducted a study aimed at participants’ daily physical activity and daily satisfaction with life using a sample comprised of emerging adults. Their results showed that emerging adults reported higher daily satisfaction with life when they had regular physical activity. Another study surveyed older adults’ level of physical activity and their satisfaction with life.^29^ The results from the survey showed that satisfaction with life was positively related to the levels of physical activity of older adults^30^ looked into the association of changes in moderate-to-vigorous physical activity with satisfaction with life and mental health. They found that participants who reported the most significant decline in physical activity had lower life satisfaction and higher mental health problems.

Personas are commonly used in the design phase of app development and policymaking to identify the target groups and their characteristics. Generally, app developers use design tools such as personas to understand their users’ needs, and policymakers use personas to provide more people-centric services.^31^ Research surrounding the use of personas for digital health applications is limited. Duan et al.^32^ looked into the use of personas as a tool that could be used to capture the mental model of the ageing population in China and how health app developers could use the resultant findings to develop health apps aimed at the target groups. A study by Holden et al.^33^ investigated the groups within hypertensive patients and their needs using clustering analysis to identify the groups and qualitative interviews to identify their needs. Based on the personas and the needs of the personas, they created and tested a mobile health app that effectively helped hypertensive patients with self-management of their disease. Another study investigated the development of personas of older adults with recent heart failure as part of a user-centered design to understand the needs of their target segments.^34^ Another research focused on developing a framework for design that included personas to facilitate the process of developing digital health technologies based on users’ needs. Policymakers could use the framework to understand the effectiveness of digital health technologies in improving healthcare.^35^ Several studies have investigated the factors impacting user engagement based on certain features,^36^ certain populations or certain apps.^3,37^ A study looking into the criteria for young people to use mental health apps found accessibility, security, and evidence-backed mental health apps as the key important aspects that young people look for when selecting mental health apps.^38^ However, research surrounding user archetypes is limited in health informatics.

In this paper, we aim to identify the user archetypes of physical and mental health apps based on app usage and two different psychometrics of personality and satisfaction with life. The identification of user archetypes is conducted through clustering analysis that takes the personality traits of extraversion and neuroticism as features for the mental health apps and the personality traits of conscientiousness and neuroticism as the features for the physical health apps.

## Method

### Dataset

The dataset was collected through SPACE: Break phone addiction app, an Android app that helps users monitor their phone usage. Users who installed the app were first asked for their consent to participate in this research. For those who consented to participate in this research, the app collected their demographics and psychometrics such as personality and satisfaction with life through a designated survey (Space Smartphone Wellbeing Research, BFI-10, and SWLS). The collected data included each app session, along with the name of the app and the time stamps of the start and end of the session. The app privacy policy, to which all users consented, includes the consent to share phone usage data anonymously with academic partners to use for research on digital wellbeing. Nonetheless, the app required users’ explicit consent before collecting and utilizing their data for this research. Those who consented were offered a premium version of the app as a reward. The participants included in the research were limited to the age group of 18 years and above. The dataset contained 602 participants from ten different countries: Sweden, Australia, Netherlands, Canada, Germany, India, United Kingdom, Brazil, France, and United States. We chose these ten different countries since the majority of the users of the app were from these countries. We wanted to minimize the possibility of getting only a few participants from other countries where the app was not popular. The data collection for the dataset was done from October 2020 to April 2021.

### Data preparation

The dataset recorded the apps of each user however, it did not collect information on the category of the app. Google Play Store has 49 app categories^39^ and using the Google Play categorization, the app categories and descriptions were extracted for each app recorded in the dataset. Table 1 shows a sample of the extracted app categories and descriptions.

**Table 1.** Sample of app categories and their descriptions.

Apps	Title	Category	Category Id
Twitter	Twitter	SOCIAL	C44
WhatsApp	WhatsApp Messenger	COMMUNICATION	C7
Facebook	Facebook	SOCIAL	C44
9GAG	9GAG: Funny gifs, pics, fresh memes and viral videos	ENTERTAINMENT	C10
CoronaMelder	CoronaMelder	MEDICAL	C36
MyFitnessPal	Calorie Counter—MyFitnessPal	HEALTH_AND_FITNESS	C31

The app categories extracted by the Google Play categorization were based on the developer's choice and hence, a few apps were miscategorized. The dataset contained around 800 apps out of which the health and medical-related apps were extracted. Health and fitness apps contained apps related to personal fitness, workout tracking, health, and safety, while medical apps contained apps related to clinical references, clinical apps, and medical journals, amongst others. We extracted apps from the health and fitness and medical categories along with the leading apps used in productivity and lifestyle categories. Additionally, keywords such as “health,” “fitness,” “medical,” and “disease” were searched in the description of the apps to ensure all health and medical-related apps were extracted from the dataset. Almost 200 apps were recategorized. For this study, after extracting the health and medical apps, the extracted apps were manually checked to classify them into the physical health and mental health app categories. To classify the apps into physical health and mental health, three of the authors classified individually a set of apps constituting 10% of the total number of apps. The authors met and discussed a unified procedure for classifying the remaining apps. The first author then classified the remaining apps based on the agreed procedure. In case of uncertainty in the app category, a senior author was invited to scrutinize and make the final decision.

Of these 690 health- and medical-related apps, we identified 115 mental health-related apps and 205 physical health-related apps. The inclusion criteria for mental health apps were apps based on online therapy, mindfulness, meditation, wellbeing, and mental health disorders. Examples of such apps are Wysa,^40^ Headspace,^41^ and Calm.^42^ The inclusion criteria for the physical health apps were apps based on workouts, nutrition, water trackers and reminders, and diet trackers. For example, apps that met the inclusion criteria include MyFitnessPal,^43^ Plant Nanny,^44^ and Fitbit.^45^

### Measures

The usage of mental and physical health app users was measured through the average daily launches of the apps for the total number of days. This is because, with users’ engagement with health apps found to be minimal, the average daily launches may be used to show their intent to seek help and hence, is used to represent the average usage. The users with less than a day of usage were removed from both datasets of mental health and physical health apps to account for the possibility that the users may have installed the app for trial and uninstalled it after facing technical issue or finding it difficult to use. Additionally, those with usage less than a day mostly had an average number of launches below 1.

Big Five Inventory – 10 (BFI-10). The personality types of neuroticism, extraversion, and conscientiousness were measured through the BFI-10 personality test The BFI-10 is an abbreviated version of the BFI-44. Instead of the 44-items of the BFI-44, the BFI-10, with a reliability measure of 0.75, uses ten items to measure the Big Five dimensions of extraversion, agreeableness, conscientiousness, neuroticism, and openness.^46^ The BFI-10 maintains the high reliability and validity of the original BFI-44. Extraversion is described as the degree of sociability, boldness, and talkativeness. Agreeableness refers to being helpful, understanding, and supportive toward others. Conscientiousness is defined as being organized, disciplined, and goal oriented. Neuroticism is displayed through the degree of emotional stability, anxiety, and self-discipline. Openness to experience refers to being open-minded, intellectually curious, and imaginative.^47^ The BF1-10 measures these five dimensions using the participants’ scores on a 5-point Likert scale, with 1 indicating strongly disagree and 5 indicating strongly agree.

*Satisfaction With Life Scale*. Satisfaction with life was measured through the SWLS. The SWLS was developed to measure global life satisfaction rather than specific domains such as health and energy.^18^ The SWLS is a 5-item validated survey measured on a 7-point Likert scale. The 7-point Likert scale is as follows: 1 = strongly disagree, 2 = disagree, 3 = slightly disagree, 4 = neither agree nor disagree, 5 = slightly agree, 6 = agree, 7 = strongly agree. The SWLS has been reported to have a reliability score of 0.87.^18^ The SWLS is measured by summing the score of all 5 items. The higher one scores on the SWLS, the higher their satisfaction with life. The SWLS score ranges from 5 to 35, with different cut-off points determining the state of satisfaction, with a score of 20 being a state of neutral. Scores within the range of 5–9 indicate the individual is extremely dissatisfied, whereas scores from 31 to 35 indicate the individual is extremely satisfied.^48^ The categorical SWLS score was encoded to ordinal data with 1: extremely dissatisfied with life, 2: dissatisfied with life, 3: neutral, 4: satisfied with life, and 5: extremely satisfied with life.

### Clustering strategy

Python 3.0 was used to implement clustering analysis. The identification of the clusters present in the data was performed using the two-phase divide and recursive merge technique. According to this technique, the maximum number of clusters is created and merged recursively to identify the distinct clusters in the dataset.^49^ The clustering analysis on users of mental health apps utilized four features: average number of launches, satisfaction with life, neuroticism, and extraversion. A low correlation was found between the four features of mental health apps. The clustering analysis performed on users of physical health apps utilized the four features: average number of launches, satisfaction with life, conscientiousness, and extraversion. The four features for physical health app analysis were found to have low correlation. Due to a mix of categorical and numerical dataset types within the dataset, the k-prototypes clustering algorithm was used.

The first phase of the divide and merge technique involves selecting the maximum number of clusters using the elbow plot. The elbow plot method is the most common method to determine the optimal number of clusters. It requires minimal background information regarding the dataset and its features.^50^ In the elbow method, the k-prototypes algorithm is run for different clusters, and the cost is calculated for each number of clusters. The k-prototypes algorithm uses either random initialization mode or manual setting mode to determine the initial cluster centers. The use of either method of initial cluster selection leads to instability of the clustering results produced by this algorithm.^51^ To counter the instability of the k-prototypes algorithm, the algorithm is run ten times on the different numbers of clusters ranging from 1 to 20. Furthermore, outliers may impact the choice of the initial cluster center thereby, reducing the performance of the clustering analysis.^52^ Therefore, based on boxplot graphs, the users whose daily launches were significantly different from other participants were removed. The cost, which is the sum of all dissimilarities between the clusters, is averaged and plotted. The maximum number of clusters is chosen when the curve follows an almost linear direction.

The second phase of the divide and merge technique involves recursively merging the clusters until considerably distinct clusters are found. The recursive merge is executed using the *t*-test. The *t*-statistic value is calculated for the centroids of each cluster pair and used to determine whether the merge between the cluster pairs should occur. While the *t*-test also calculates the *p*-value of the cluster pairs, the *p*-value is not used since it helps to identify whether the cluster centroids are significantly different but does not provide enough evidence on whether the centroids are similar. The clusters’ centroids were taken as the average of the mean and mode of the clusters to get the best representation of the cluster's center. The *t*-statistic value, the distance between two cluster centroids, is plotted against the cluster pairs to get an elbow plot based on the distances between the clusters. The threshold value is determined based on the “elbow” of the plot. Distances between the cluster centroids below the threshold value are merged, and then the *t*-statistic is recalculated, and the clusters are merged until distinct clusters are found. Furthermore, we also conduct a sensitivity analysis to assess the robustness of the results. Two different techniques were used to study robustness: robustness toward suspected outliers and robustness toward changing the number of clusters. For outliers’ robustness, suspected outliers are reintroduced to the sample and the results are then rerun to evaluate the algorithm. Additionally, we change the number of clusters originally produced in order to study the difference in results produced by the new clusters.

### Mental health apps clustering analysis

**Figure 1.**
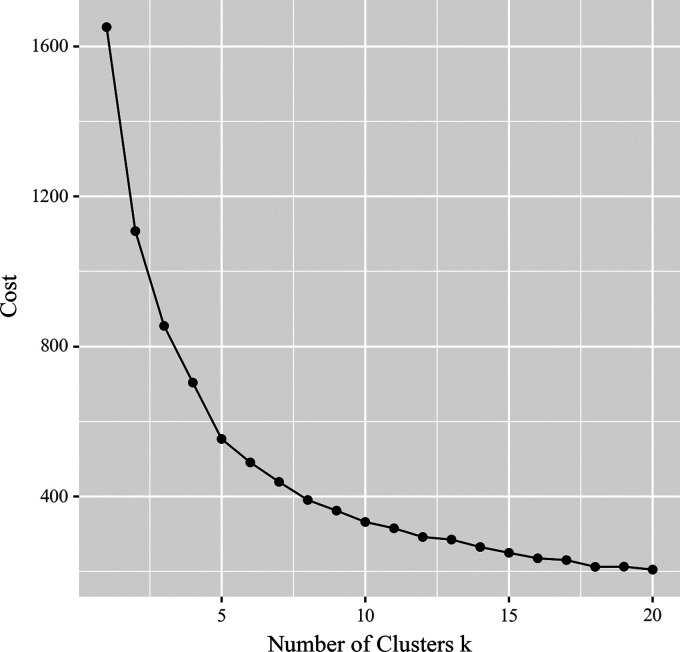
Maximum number of clusters of mental health apps users with elbow plot.

The elbow plot achieved from running the clustering analysis on the mental health apps usage dataset is shown in Figure 1. Based on the elbow plot, the maximum number of clusters was chosen as 10.

**Figure 2.**
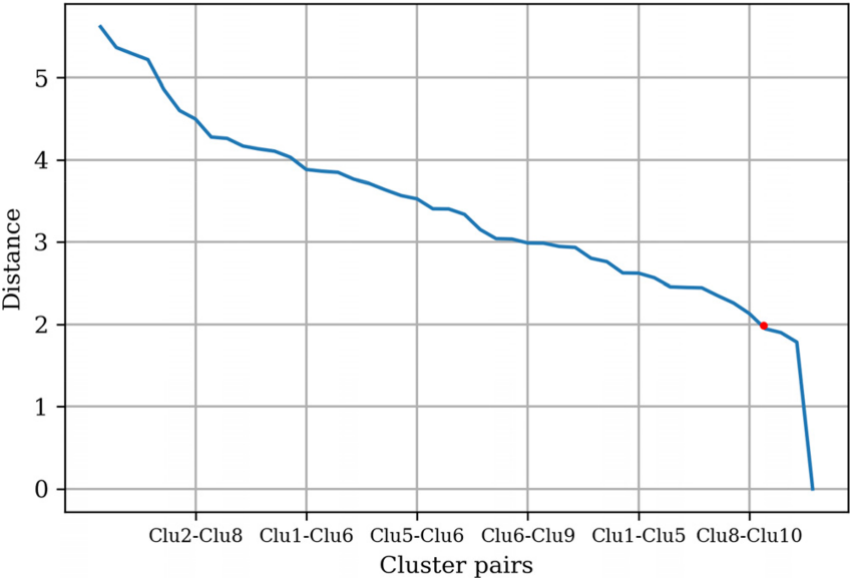
Distances between centroids pair based on 10 mental health app clusters.

The *t*-statistic between the cluster pairs was calculated, and the distances between the centroids of clusters were plotted as shown in Figure 2. The threshold value of approximately 2 was chosen based on the “elbow” found on the plot. Clusters with a *t*-statistic below the threshold were merged.

**Figure 3.**
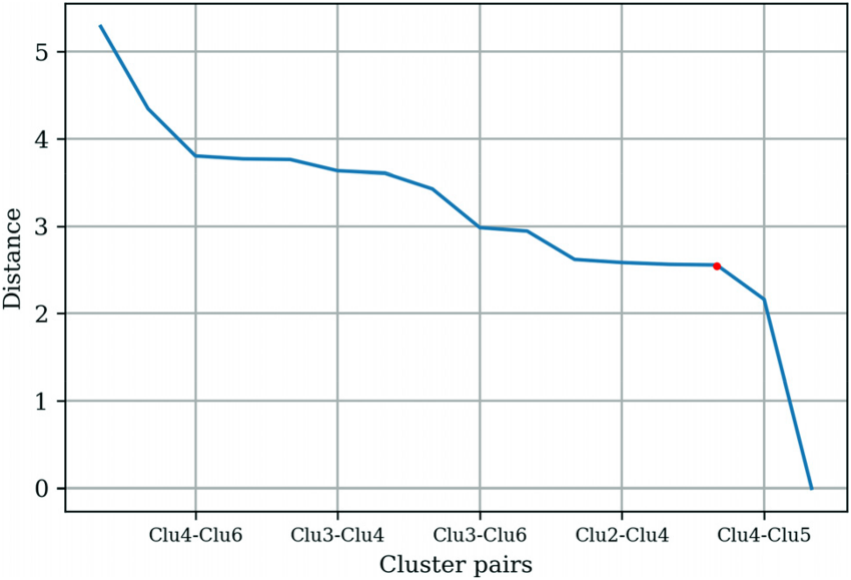
Distances between centroids pair based on six mental health app clusters.

Clusters 2 and 6 were merged based on the threshold value, and Clusters 4, 7, 8, and 10 were merged. The *t*-statistic between the cluster pairs was recalculated, and the distances between the centroids of the clusters were replotted, as shown in Figure 3. The threshold value of approximately 2.5 was selected based on the “elbow” found on the plot. Clusters with a *t*-statistic below the threshold were merged. Clusters 4 and 5 were merged with Cluster 1 based on the threshold value, and four distinct clusters were found.

### Physical health apps clustering analysis

**Figure 4.**
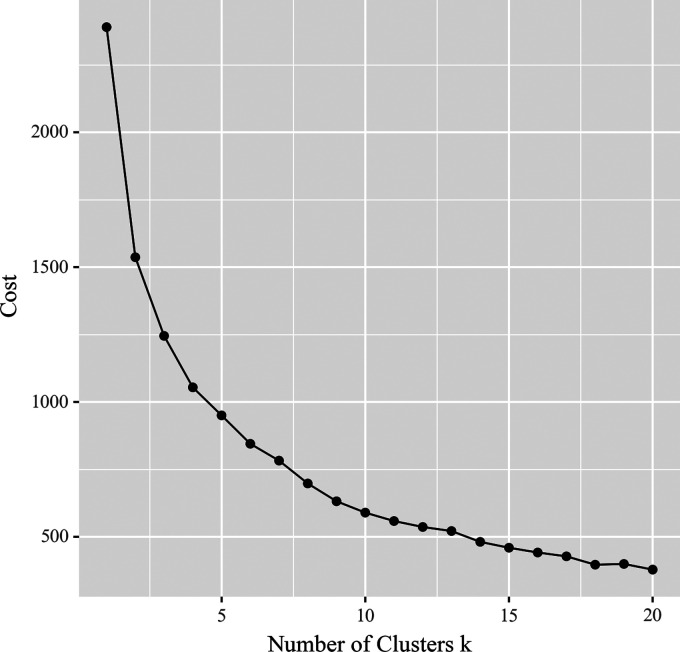
Maximum number of clusters of physical health apps users with elbow plot.

The clustering analysis on the physical health apps usage dataset produced the elbow plot, as shown in Figure 4. Based on the elbow plot, the maximum number of clusters was chosen as 10.

**Figure 5.**
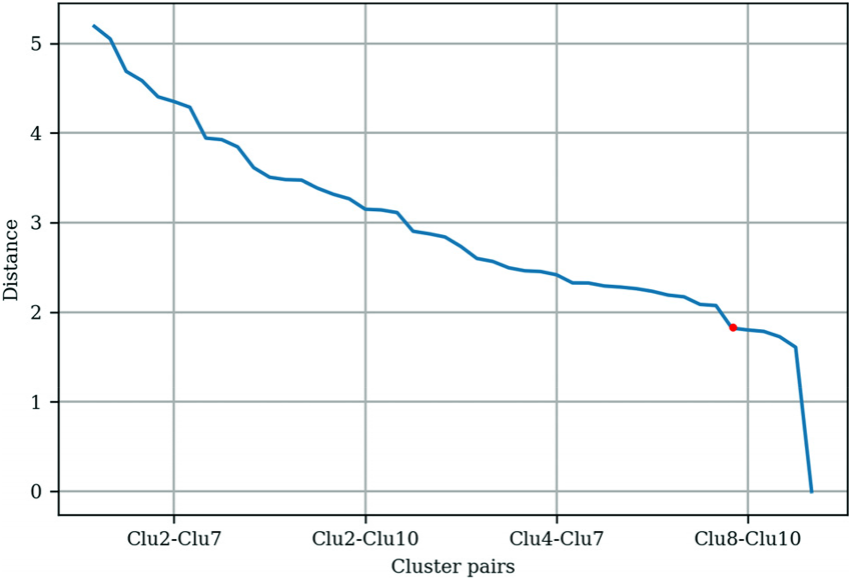
Distances between centroids pair based on ten physical health app clusters.

The *t*-statistic between the centroids of the cluster pairs was calculated and plotted as shown in Figure 5. The threshold value of approximately 1.8 was chosen based on the “elbow” found on the plot. Clusters with a *t*-statistic below the threshold were merged.

**Figure 6.**
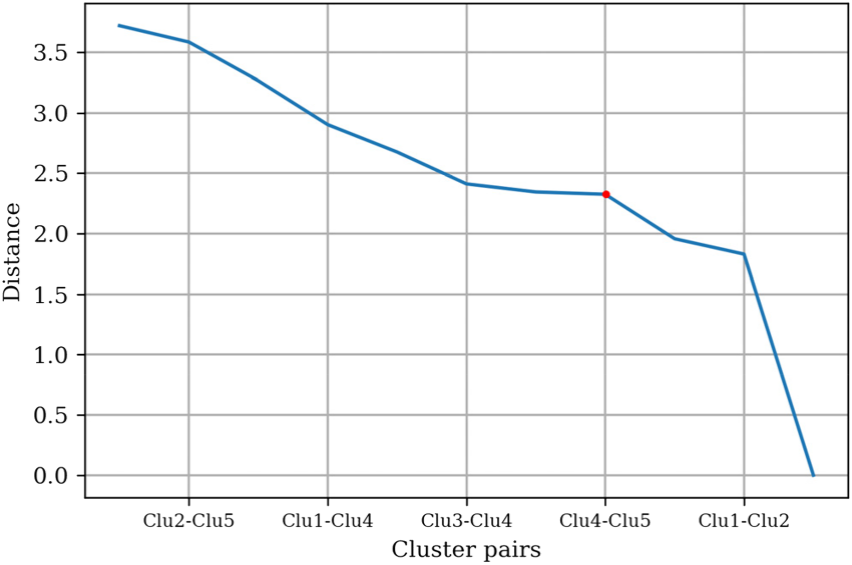
Distances between centroids pair based on five physical health app clusters.

Based on the threshold value, Clusters 2 and 6 were merged, and Clusters 3, 7, 8, 9, and 10 were merged. The *t*-statistic between the cluster pairs was recalculated and replotted, as shown in Figure 6. Two threshold values of approximately 2.4 and 1.8 were found; however, to merge the maximum number of clusters, the threshold value of approximately 2.4 was selected. Clusters with a *t*-statistic below the threshold were merged. Clusters 4 and 5 were merged, and Clusters 1 and 2 were merged based on the threshold value, and three distinct clusters were found.

## Results

### Descriptive statistics

**Figure 7.**
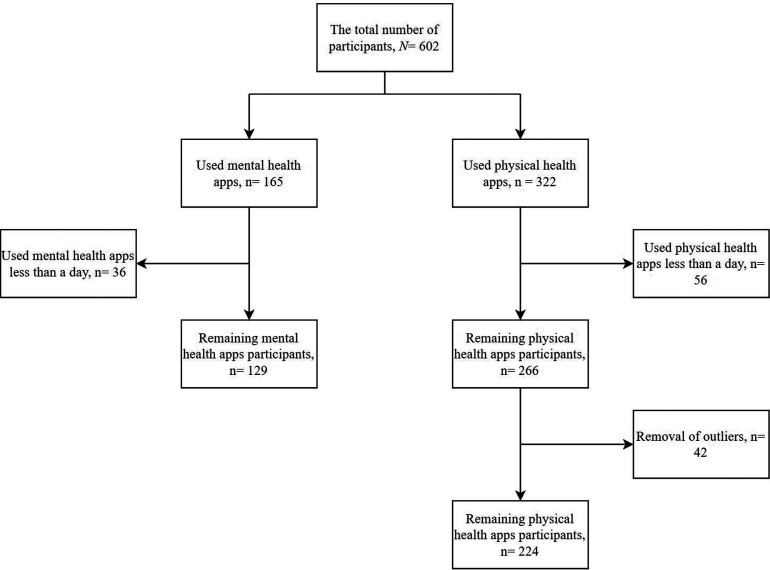
Flowchart of participants’ selection.

Two datasets were created from the original dataset to study the mental and physical health app users. The first dataset contained only the mental health app users. In total, 165 users of the 602 participants used mental health apps. Out of these 165 users, 36 users had less than a day of usage of mental health apps. These users were removed from the dataset bringing the total number of mental health app users to 129. The second dataset contained only physical health app users. In total, 322 users had physical health app usage. The removal of users with less than a day of usage brought the total number of physical health apps users to 266 users. The physical health apps dataset also contained outliers which were removed to improve the performance of the clustering analysis. Therefore, the final dataset contained 224 users of physical health apps. Few users had both mental and physical health app usage and were accounted for in both datasets. Figure 7 shows the flowchart for the participants’ selection.

**Figure 8.**
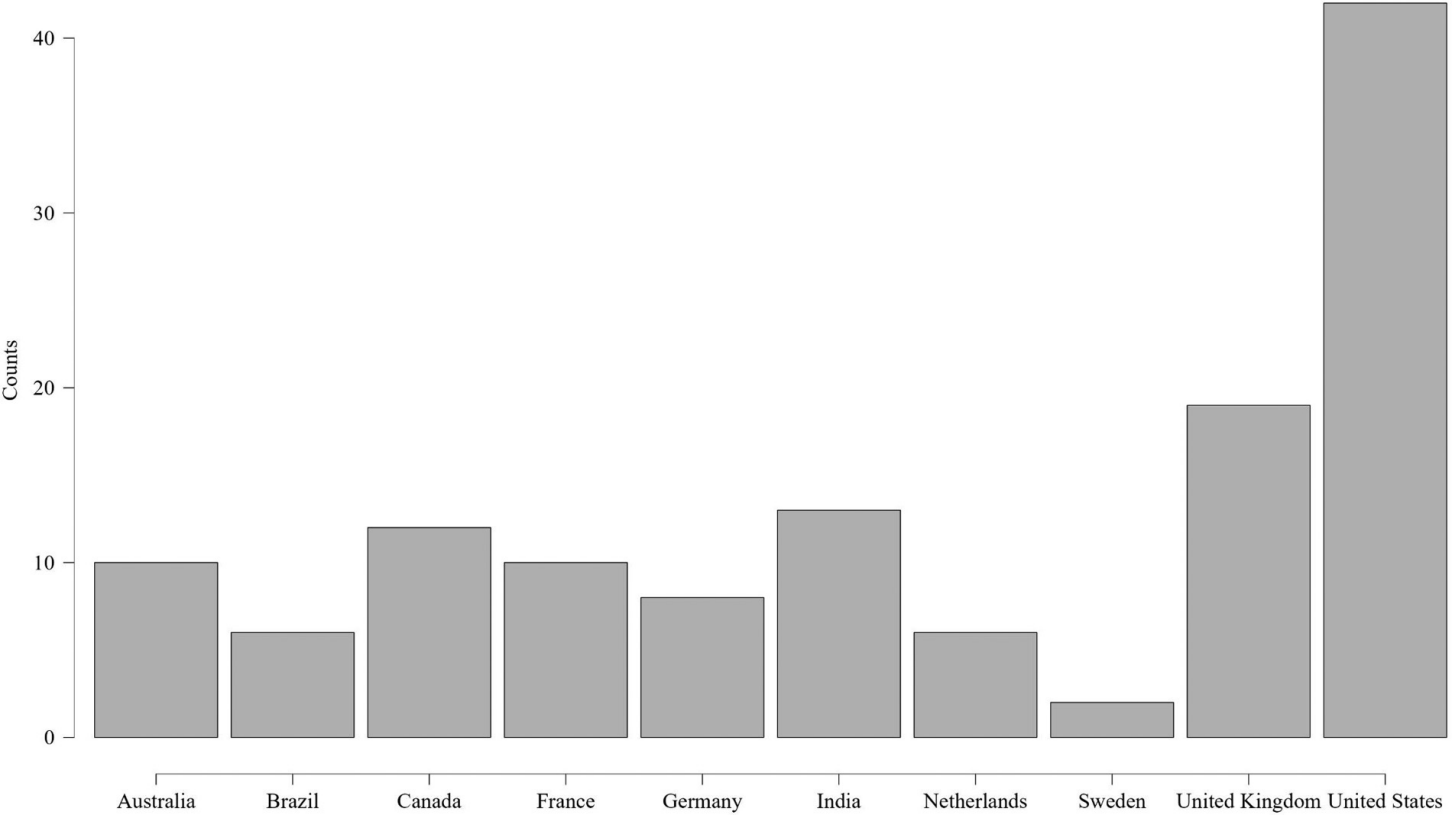
Distribution of mental health apps participants based on country.

Table 2 summarizes the descriptive statistics of the sample of mental health apps. Mental health apps had 129 participants, out of which 62% were females. Age was classified into two categories of emerging adults and adults. UNICEF classified emerging adults as 15–24 years whereas adults are above 24 years.^53^ Since our study limited the participants to those above the age of 18 years, users between 18 and 24 years were classified as emerging adults and those above 24 years as adults. Mental health apps had almost 59% of participants who were adults whereas almost 41% were emerging adults. The distribution of the participants of mental health apps based on country is shown in Figure 8.

**Table 2.** Descriptive statistics of clustering features of mental health apps.

Variables	Mean	Std. Dev.
Average daily launches	1.25	1.59
Extraversion	5.81	2.17
Neuroticism	6.99	2.20
	Median	IQR
SWLS	4	2

SWLS: Satisfaction With Life Scale; IQR: interquartile range.

**Figure 9.**
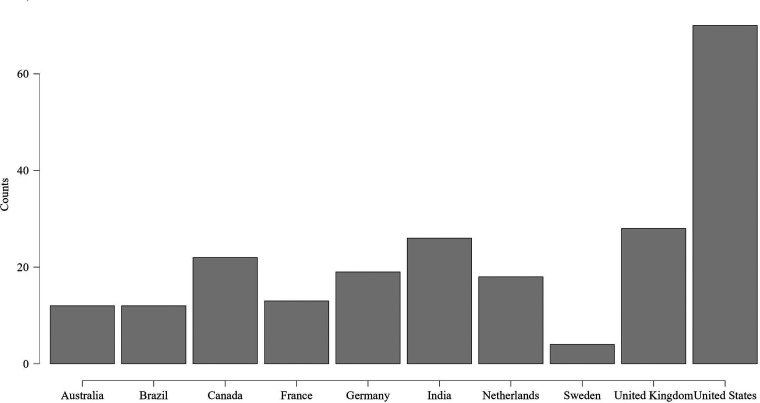
Distribution of physical health apps participants based on country.

Table 3 summarizes the descriptive statistics of the sample of physical health apps. Among the 224 participants for physical health apps, around 63% were females, and about 54% were adults. Additionally, approximately 46% were emerging adults in the sample. Figure 9 shows the distribution of the participants of physical health apps based on country.

**Table 3.** Descriptive statistics of clustering features of physical health apps.

Variables	Mean	Std. Dev.
Average daily launches	1.12	1.20
Conscientiousness	6.32	1.87
Neuroticism	6.76	2.22
	Median	IQR
SWLS	4	2

SWLS: Satisfaction With Life Scale; IQR: interquartile range.

Table 4 shows the distribution of the average launch of mental health apps of the participants based on country whereas Table 5 shows the distribution of the average launch of physical health apps of the participants based on country. The distribution of both physical and mental health apps shows that the average launches for each country are not particularly different. This may imply the lack of deviation present within the sample.

**Table 4.** Distribution of average daily launch of mental health apps by country.

	Average daily launch									
	Australia	Brazil	Canada	France	Germany	India	Netherlands	Sweden	United Kingdom	United States
*n*	10	6	12	10	8	13	6	2	19	42
Mean	0.619	0.605	1.393	1.122	0.809	1.918	1.488	0.925	1.101	1.397
Std. Deviation	0.742	0.760	1.784	1.584	0.808	2.218	2.337	1.138	1.098	1.755
Minimum	0.040	0.130	0.020	0.080	0.140	0.110	0.070	0.120	0.020	0.020
Maximum	1.990	1.990	5.470	5.380	2.350	5.800	6.210	1.730	3.740	6.900

**Table 5.** Distribution of average daily launch of physical health apps by country.

	Average daily launch									
	Australia	Brazil	Canada	France	Germany	India	Netherlands	Sweden	United Kingdom	United States
*n*	12	12	22	13	19	26	18	4	28	70
Mean	1.115	1.015	1.278	1.564	0.546	0.865	1.677	1.093	1.206	1.077
Std. deviation	1.344	1.098	1.317	1.170	0.407	1.154	1.526	1.221	1.235	1.193
Minimum	0.060	0.110	0.020	0.040	0.040	0.030	0.140	0.350	0.140	0.050
Maximum	3.820	3.540	4.080	3.440	1.430	4.280	4.870	2.910	4.550	4.760

### Mental health clusters

**Figure 10.**
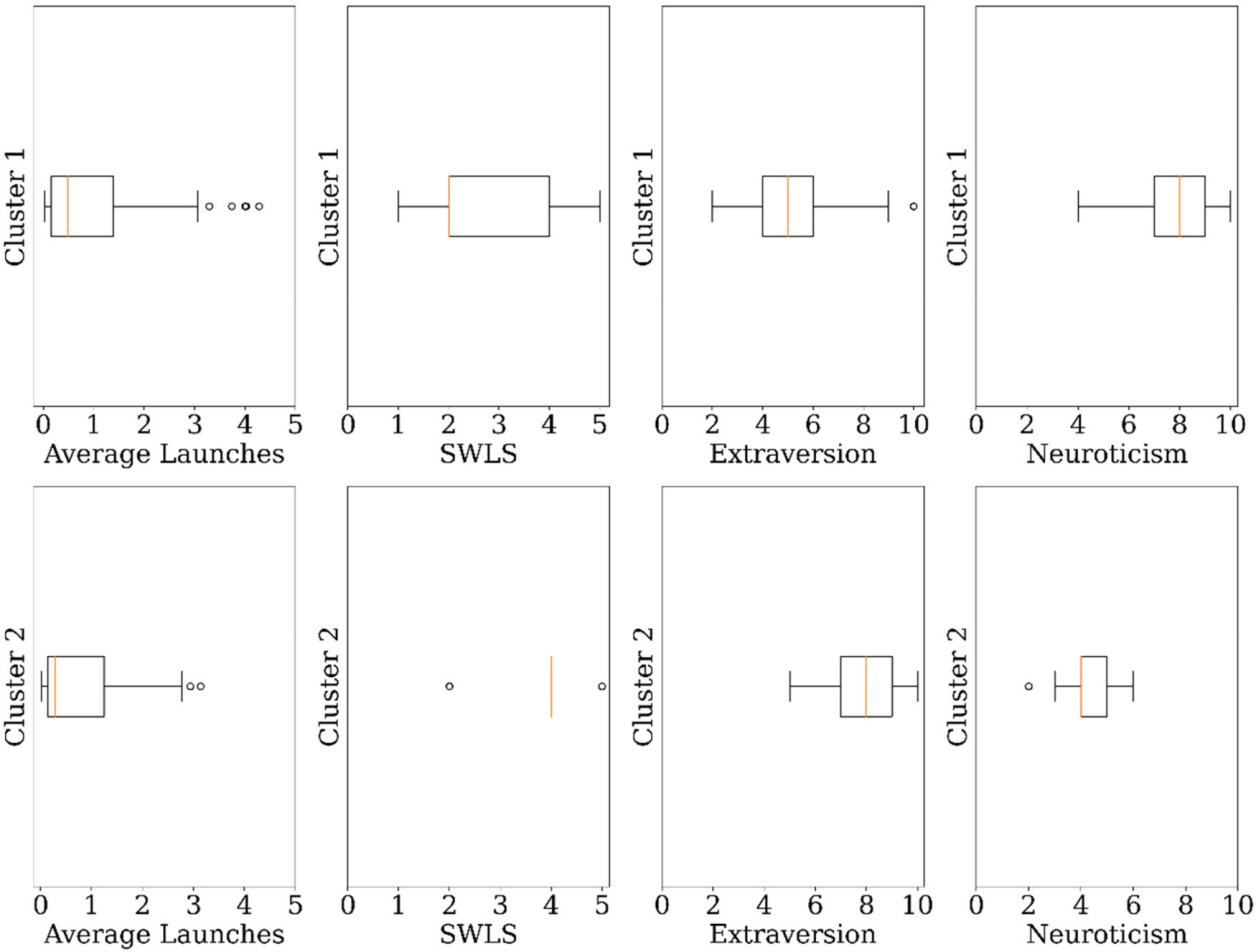
Boxplots of the features of mental health app clusters.

The clustering analysis identified four clusters in the sample of mental health app users which are Cluster 1 (*n* = 88, 68%), Cluster 2 (*n* = 32, 25%), Cluster 3 (*n* = 5, 3.9%), and Cluster 4 (*n* = 4, 3.1%). Clusters 3 and 4 were discarded from the sample due to their comparatively small sizes, implying that they are outliers. Figure 10 shows the boxplots of the features of the final clusters. Cluster 1 (*help-seeking users*) has users with low satisfaction with life (median = 2.0, interquartile range (IQR) = 2.0), moderate extraversion (median = 5.0, IQR = 2.0), and high neuroticism (median = 8.0, IQR = 2.0). Users in Cluster 1 have an average daily launch of almost once a day. Cluster 2 (*maintenance users*) has users with high satisfaction with life (median = 4.0, IQR = 0.0), high extraversion (median = 8.0, IQR = 2.0), and low neuroticism (median = 4.0, IQR = 1.0). Users in Cluster 2 have sporadic daily usage of mental health apps.

### Physical health clusters

**Figure 11.**
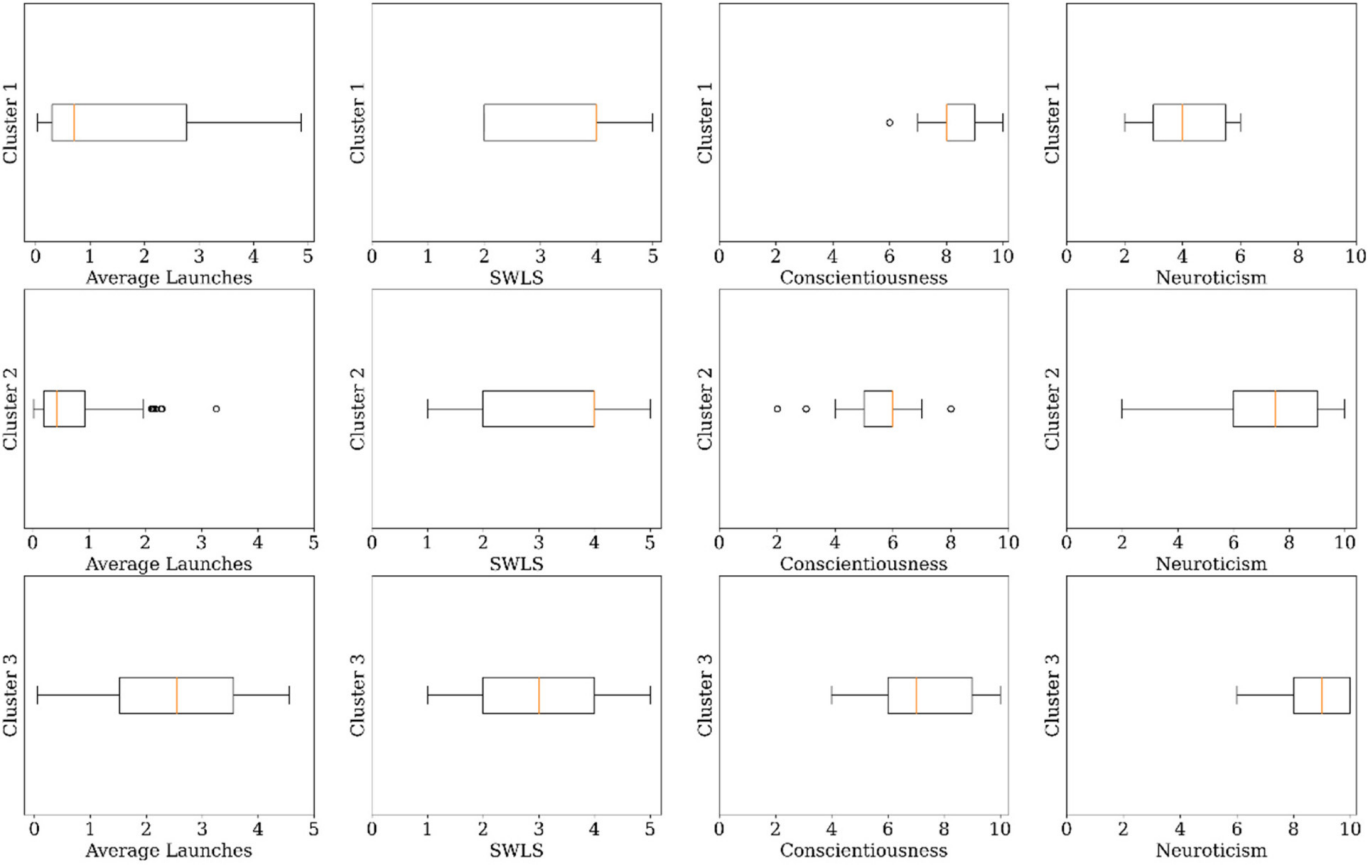
Boxplots of the features of physical health app clusters.

The clustering analysis identified three clusters in the sample of mental health app users which are Cluster 1 (*n* = 51, 22.8%), Cluster 2 (*n* = 140, 62.5%), and Cluster 3 (*n* = 33, 14.7%). Figure 11 shows the boxplots of the features of the final clusters. Cluster 1 (*happy conscious occasional users*) has users with high satisfaction with life (median = 4.0, IQR = 2.0), high conscientiousness (median = 8.0, IQR = 1.0), and low neuroticism (median = 4.0, IQR = 2.5). Users in Cluster 1 have an average daily launch of almost once a day. Cluster 2 (*happy neurotic occasional users*) has users with high satisfaction with life (median = 4.0, IQR = 2.0), moderate conscientiousness (median = 6.0, IQR = 1.0), and high neuroticism (median = 7.5, IQR = 3.0). Users in Cluster 2 have an average daily launch of almost one launches a day. Cluster 3 (*neutral neurotic frequent users*) has users with moderate satisfaction with life (median = 3.0, IQR = 2.0), high conscientiousness (median = 7.0, IQR = 3.0), and high neuroticism (median = 9.0, IQR = 2.0). Users in Cluster 3 have an average daily launch of about three launches a day.

### Sensitivity analysis results

**Figure 12.**
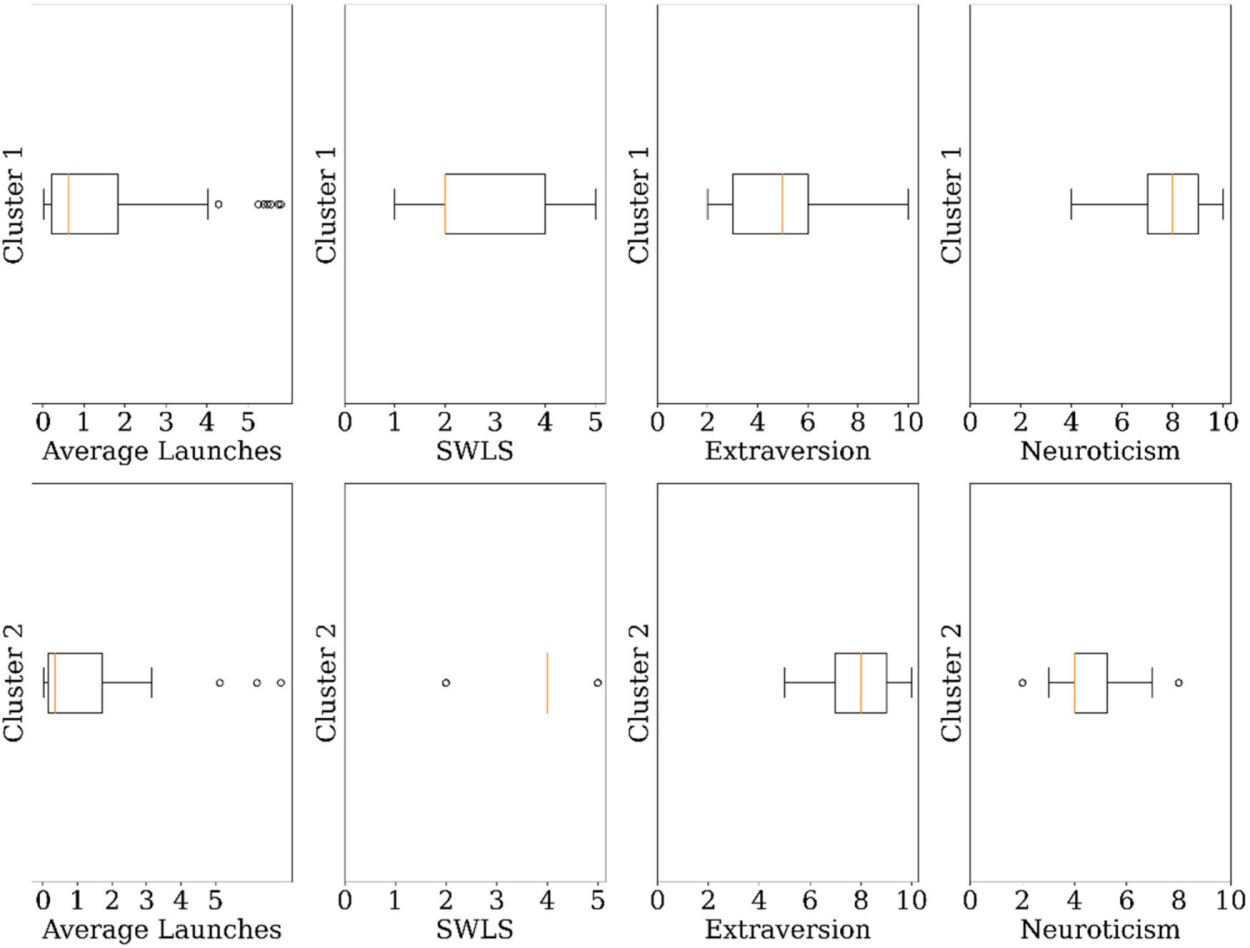
Mental health clusters with outliers.

The robustness of the algorithm toward the clusters results is also studied. The k-prototype algorithm is sensitive only to outliers for initial cluster centers whereas for later stages it is robust to outliers. For example, on re-running the algorithms with the suspected outliers for mental health apps, the clusters produced mirrored the two clusters found previously. Figure 12 shows the results of the mental health clusters found with the suspected outliers and which are in close proximity to the previous results found.

We also checked the robustness of our clustering outcomes by changing the number of clusters. For example, for the mental health apps, we ran the analysis again with three clusters and achieved a result almost similar to the previously obtained results.

## Discussion

The study aimed to identify the user archetypes of mental and physical health apps based on usage of the apps, personality, and satisfaction with life. User archetypes and personas can be a crucial methodological phase in health app development to better user engagement^32^ and understand user behavior.^54^ Health apps that are personalized and customized to users help to improve user engagement.^55^

For mental health apps, two different user archetypes were identified. The *help-seeking* user archetype has users who launch mental health apps almost daily. These users are dissatisfied with their lives, moderately extraverted, and highly neurotic. The study done byShokrkon and Nicoladis^14^ found neuroticism to be negatively associated with mental health and extraversion to be positively related to mental health. Additionally, poor mental health is associated with low levels of satisfaction with life.^17^ This may explain the usage of help-seeking user archetypes since, with high neuroticism and moderate extraversion, they may be suffering from low mental health and, thereby, low satisfaction with life.

The mental health apps users of the *maintenance* archetype are satisfied with life, extraverted, and emotionally stable. The average daily launch of *maintenance* user archetypes was infrequent, suggesting they may not regularly launch mental health apps. Extraversion and emotional stability are positively associated with mental health.^56^ *Maintenance* users are high in extraversion and emotional stability and may not be dealing with mental health problems and are satisfied with life.^57^ Prochaska and Velicer reported that users tend to use mental health apps when they feel the need for them. Additionally, behavioral change theory states that people tend to reduce their frequency of use of behavioral change support tools when they are aiming to maintain their behavior while not reverting to old behaviors.^58^ This may also be why they do not regularly use mental health apps as they may not feel the need for it.

Three different user archetypes were identified for physical health apps. *Happy conscious occasional* user archetypes have users who are satisfied with life, high in conscientiousness, low in neuroticism, and they launch physical health apps almost once every day. Research shows high satisfaction with life,^59^ high conscientiousness, and low neuroticism predict improved physical health.^23^ Additionally,^25^ reported conscientiousness to have a positive relationship with the usage of physical health apps. This may explain why *happy conscious occasional* users may launch physical health apps regularly to ensure that their physical health is maintained. People high in conscientiousness also tend to adopt healthy behaviors.^60^ Physical health apps can provide behavioral change support to users^2^ and *happy conscious occasional* users may launch them regularly to track their health behaviors.

*Happy neurotic occasional* user archetypes have users who are satisfied with life, moderate in conscientiousness, high in neuroticism, and launch physical health apps regularly. Neuroticism is often negatively associated with physical health.^23,61^ However, neuroticism may also promote the adoption of healthy behaviors since worrying, and distressing may push them toward healthy behavioral choices.^62^ The regular use of physical health apps may imply that *happy neurotic occasional* users are adopting healthy behaviors in order to maintain their physical health. Their high satisfaction with life may also be because they are focused on improving physical health and adopting healthy behaviors.

*Neutral neurotic frequent* user archetypes comprise users who are neither satisfied nor dissatisfied with life and score high on conscientiousness and neuroticism. *Neutral neurotic frequent* users launch physical health apps almost three times a day. Highly neurotic people tend to be concerned about their health, while highly conscious people tend to do something about their health concerns, such as adopting healthy behaviors.^63^ This may explain the need for these users to launch physical health apps as they may be seeking behavioral change support aimed at their physical health. Additionally, physical health is linked to satisfaction with life.^59^ The use of physical health apps for behavioral change may indicate that they are trying to improve their physical health and are neither satisfied nor dissatisfied with life.

The study has a few limitations. The sample size of health apps was sufficient to perform the clustering analysis on mental and physical health apps, according to Nisha et al.^64^; however, it limited the ability to study further the user archetypes based on the subcategories of mental and physical health apps. The subcategories of mental and physical health may help to understand whether certain subcategories such as mindfulness-based or tracking-based apps interacted with personality and satisfaction with life. Another limitation is that the study does not take into account the discontinuation of health apps. The limitation comes from the restriction by Android to know when users uninstall an app and to gather information regarding their reasons for departure. Users tend to discontinue the use of health apps while initially downloading them.^65^ The average daily launches represent the use of health apps on a daily basis but looking at the discontinued use along with the average daily launches may help to identify the users who left the app and whether a certain personality or state of life satisfaction influences the discontinued use. The data on discontinued use may have helped to include an additional feature in the clustering analysis that would identify users who benefited from health apps and those who discontinue the use of health apps due to lack of benefit. Another limitation of the study is the possibility of an information bias that may occur due to the app being only in the English language thereby, the questionnaire and survey presented in the English language. While the wording of all questions and scales used was relatively simple, participants not particularly well versed in English may find it hard to complete the survey. However, it is worth noting that participation in the study was voluntary and participants were free to leave the survey at any point. Additionally, with the aim of the app to inform users of their phone usage, the app was installed by users who wanted to be aware of their phone usage behaviors. Hence, the results have to be interpreted with caution as this may introduce a selection bias in the sample with the majority of the users participating to change or be aware of their phone usage.

Personality and satisfaction with life tend to impact a user's decision to adopt smartphone apps.^66,67^ Theoretical implications from the findings of this study show how usage of health apps, personality, and satisfaction with life interact with each other. The findings of this study help to identify the users who use health apps occasionally and those who use them frequently based on personality and satisfaction with life. The results from this study also have practical implications. The role of user archetypes is important in app development to ensure user acceptance; however, limited research has looked into user archetypes in health informatics.^32^ Identifying user archetypes that use health apps may help app developers understand who uses health apps and thereby may help increase user engagement and retention. Apps that provide tailored help and support to their users have higher user adherence and lasting effects.^68^ A meta-analysis stated that people high in neuroticism tend to be highly motivated to seek treatment for mental health.^12^ The launch of mental health apps by *help-seeking* users on a daily basis may be due to their motivation to seek support. Designers of health apps may need to take into account the motivation of the use of *help-seeking* users when designing health apps. Moreover, designs for *help-seeking* users may need to include personalized content to maintain their motivation. An online survey found users are motivated to find health-related sites when suffering from health concerns.^69^ Further, as their health improves, they tend to shift from treatment-related content to prevention-related content. The sporadic use of *maintenance* archetype, thereby, may be due to their shift toward prevention-related content and hence, health apps design may need to take this into account. For physical health archetypes, design implications for *happy conscious occasional* users may need to consider the use of goal settings and activity logging since the regular launch of health apps may be to ensure their physical health is maintained and tracked. Furthermore, *happy neurotic occasional* users may feel the need for guidance-based physical health content along with logging content since high neuroticism may push them toward adopting healthy behavioral choices. *Neutral neurotic frequent* user archetypes may benefit from a design that gives positive feedback and encouragement as these users are conscious about their health but neutrally satisfied with life.

Furthermore, the findings of this study can be used by policymakers to understand the users of health apps and invest in health technologies relevant to the populations’ needs. The archetypes of health apps also show the need to regulate the use of health apps by policymakers. Current health apps are developed mainly by developers without involving health professionals, and regulations on these health apps are rarely placed.^70^ With user archetypes such as *maintenance* users using these apps when they feel the need for it, regulations may help to ensure the users are provided with the proper help they are seeking that would improve their health rather than deteriorate it. Furthermore, while these apps may be used to provide support, they cannot replace the involvement of a health professional and cannot claim that the involvement of a health professional is not needed^71^. Hence, regulations in place are needed to ensure data transparency and accuracy are practiced.

Future work in this area may focus on studying the user archetypes who left health apps and whether they left due to improved conditions or disinterest in the health apps. The type of apps these users use can also be studied to understand the categories of health apps with the most dropouts. Additionally, mental health and physical health apps have further subcategories and future work may investigate the relationship between the user archetypes and app subcategories. This could help to identify and understand whether different app types are associated with different user archetypes. Moreover, future work may include studying user archetypes based on demographics such as age and gender to study the relationship between the demographics and the user archetypes and whether a certain user archetype is predominant in a certain demographic.
